# Correction: Šoša et al. Metabolic Dysfunction-Associated Steatotic Liver Disease Induced by Microplastics: An Endpoint in the Liver–Eye Axis. *Int. J. Mol. Sci.* 2025, *26*, 2837

**DOI:** 10.3390/ijms27177645

**Published:** 2026-08-26

**Authors:** Ivan Šoša, Loredana Labinac, Manuela Perković

**Affiliations:** 1 Department of Anatomy, Faculty of Medicine, University of Rijeka, 51000 Rijeka, Croatia; 2 Department of Pathology and Cytology, General Hospital Pula, 52100 Pula, Croatia; loredana.labinac@obpula.hr (L.L.); perkovic.manuela@gmail.com (M.P.)

In the original publication [1], citation reference 5 was retracted. To enhance the quality of the manuscript, we decided to replace the previous reference number 5 from the original manuscript with the new reference [2] at the same position in the reference list.

Original reference number 5: Mirani, A.; Kianfar, E.; Maleknia, L.; Javanbakht, M. Recent advances in nicotine electrochemical biosensors: A review. *Case Stud. Chem. Environ. Eng.* **2024**, *9*, 100753.

New reference number 5: Yin, M.; Lin, H.; Zhang, L.; Wei, X.; Sun, Y.; Luo, Y.; Yang, H.; Deng, C.; Xu, D. Antibody-assisted MIL-53(Fe)/Pt-based electrochemical biosensor for the detection of the nicotine metabolite cotinine. *Bioelectrochemistry* **2023**, *153*, 108470, https://doi.org/10.1016/j.bioelechem.2023.108470. 

The overall numbering and ordering of the references remain unchanged.

The authors state that the scientific conclusions are unaffected. This correction was approved by the Academic Editor. The original publication has also been updated.
